# The Influence of FTO Polymorphism rs9939609 on Obesity, Some Clinical Features, and Disturbance of Carbohydrate Metabolism in Patients with Psoriasis

**DOI:** 10.1155/2019/7304345

**Published:** 2019-01-13

**Authors:** Małgorzata Tupikowska-Marzec, Katarzyna Kolačkov, Aleksandra Zdrojowy-Wełna, Natalia K. Słoka, Jacek C. Szepietowski, J. Maj

**Affiliations:** ^1^Department and Clinic of Dermatology, Venereology and Allergology, Wroclaw Medical University, Poland; ^2^Department and Clinic of Endocrinology, Diabetology and Isotope Therapy, Wroclaw Medical University, Poland

## Abstract

**Background:**

Psoriasis is often accompanied by obesity, hyperlipidemia, diabetes, and metabolic syndrome as risk factors of cardiovascular conditions and premature mortality.

**Objective:**

The study was aimed at investigating whether psoriatic patients, who carry risk allele of obesity-related* FTO* gene, are more predisposed to obesity and metabolic disturbances and whether it influences the severity of psoriasis.

**Methods:**

197 patients with psoriasis, representing Lower Silesia region of Poland, underwent physical examination and anthropometric measurements. Blood samples for biochemical and genetic analysis were collected. All patients were genotyped for* FTO* gene rs9939609 variant. Identification of SNP was conducted with the use of minisequencing method.

**Results:**

Around 63% of patients were carriers of at least one risk allele A and 20% were AA homozygotes. The A allele was associated with increased BMI and hip and waist circumferences. The carriers of risk allele had increased PASI and CRP values and tended to have an increased insulin concentration.

**Conclusion:**

Psoriatic patients, carriers of risk allele of* FTO* gene rs9939609, have an increased risk for more severe psoriasis and obesity and may develop obesity-induced insulin resistance.

## 1. Introduction

Psoriasis (PSO) is often accompanied by obesity, hypertension, diabetes mellitus, hyperlipidemia, or metabolic syndrome [1–6]. These comorbidities are risk factors for cardiovascular diseases and premature mortality [7, 8]. Until now the reasons for more frequent occurrence of obesity or metabolic disturbances in patients with PSO have not been fully understood. The main cause is lifestyle and chronic inflammation but it is possible that also genetic factors may play some role.

Genome-Wide Association Studies (GWAS) discovered that carriers of risk alleles of* Fat Mass and Obesity Associated Gene* (*FTO)* can be predisposed to obesity and metabolic disorders [9]. Other research seems to confirm these reports. The strongest association of* FTO* gene with obesity and Body Mass Index (BMI) was shown with Single Nucleotide Polymorphisms (SNPs) such as rs9930609 and rs9930506 (with risk alleles A and G, respectively) [10–13].

The* FTO* gene encodes 2-oxoglutarate-dependent nucleic acid demethylase, the protein involved in DNA repair and energy homeostasis regulation. The* FTO* mRNA is mainly expressed in the hypothalamus. Though the exact physiological function of* FTO* gene is not well known yet, it has some influence on nervous and cardiovascular systems [9].

*Coto-Segura et al. *[14] have recently published data claiming that PSO patients, homozygous for* FTO* rs9930506 G allele, had higher BMI and increased obesity risk and probably increased risk of arthritis. Up to date, there is no data concerning the impact of rs9930609 polymorphisms of* FTO* gene on obesity, BMI, or metabolic disturbances in PSO group. It seems interesting to investigate whether PSO patients, carriers of risk allele of* FTO* rs9939609 polymorphism, are more predisposed to obesity or metabolic abnormalities and whether it influences the severity of psoriasis.

The aims of our study were therefore (1) to investigate the genotype frequency of* FTO* gene rs9939609 in Polish PSO group, inhabitants of Lower Silesia; (2) to assess the relationship between the presence of risk allele and severity of PSO; (3) to examine the association of risk allele and obesity, hypertension, and some carbohydrate disturbances.

## 2. Material and Methods

### 2.1. Study Group

During the years of 2012-2014, from a total of 597 psoriatic patients hospitalized in Clinic of Dermatology, Venereology and Allergology, the single dermatological centre of Lower Silesia, 197 subjects (96 female and 101 male, ages 30-65) were enrolled in the study. The exclusion criteria were age and immunosuppressive treatment. All patients signed consent for participation in the study. This group underwent physical examinations, blood pressure measurement, and anthropometric measurements and provided blood samples for biochemical tests and genetic analysis. Their condition was diagnosed on the basis of clinical symptoms, and the severity of the disease was estimated according to the Psoriasis Severity and Area Index (PASI). This project has been approved by the Local Ethical Committee (KB-592/12).

### 2.2. Anthropometric and Laboratory Measurements

Body weight and height were measured for Body Mass Index (BMI) calculation. Based on waist and hip circumferences a Waist-to-Hip Ratio (WHR) was estimated. Patient`s arterial blood pressure was measured. Serum CRP, glucose, and insulin concentrations were determined from fasting blood. Erythrocyte sedimentation rate (ESR) test was performed with the use of EDTA-anticoagulated whole blood samples. CRP level was determined by ELISA test (Diagnostic Systems Laboratories, USA). Glucose concentration was assessed by enzymatic method (Dade Behring Marburg GmbH, Germany). Concentration of insulin was measured by means of enzyme immunoassay (Abbott, USA). Insulin resistance indicators, HOMA, FIRI, and QUICKI, were calculated as described by other authors [15–17].

### 2.3. Genotyping

Whole genomic DNA was isolated from peripheral blood leukocytes according to a protocol from commercial DNA isolation kit (Macherey-Nagel, Germany). Identification of rs9939609 of* FTO* gene was performed by means of two methods: polymerase chain reactions, PCR (using Takara Amplification Kit, Japan), and minisequencing (using SnaPshot Multiplex Kit, Thermo Fisher Scientific, USA). The reactions were carried out in the presence of specifically designed pair of primers:

  5'-CACTAACATCAGTTATGCAT-3' – forward primer
  5'-CCATTTCTGACTGTTACCTA-3' – reverse primer

Products of the reaction were separated by capillary electrophoresis in ABI PRISM®3100 Genetic Analyzer and analyzed by GeneMapper® Software Version 4.0 (Thermo Fisher Scientific, USA). The whole genotyping procedure has previously been thoroughly described [18].

### 2.4. Statistical Analysis

All values are shown as mean ± standard deviation. Continuous variables were first tested for the normal distribution with the use of the W. Shapiro-Wilk test. The hypothesis of the equality of distribution of two sample sizes was verified with the use of the U. Mann-Whitney test. For comparison of more than two independent variables Kruskal-Wallis test was used.

A p-value less than 0.05 was considered statistically significant, while p=0.05 indicates tendency to significance. The data was analyzed with the use of the STATISTICA ver. 10.0 medical package.

## 3. Results

Depending on the presence of the risk allele A, patients were divided into 3 groups: AA, TT, and AA+AT (a group with at least one A allele, treated as being the “group at risk”). The results were compared between groups: AA* vs.* TT and AA+AT* vs.* TT.

### 3.1. Genotype Frequency of FTO Gene rs9939609

19.87% patients with PSO were carriers of the two risk alleles (AA genotype). The percentage of heterozygous AT genotype was 43.05% and 37.08% of PSO-patients were carriers of TT alleles. Although men were somewhat more frequently AA homozygotes (25.23%* vs.* 16.7%) there were no statistically significant differences between distributions of genotype frequency in relation to gender. 62.92% patients were included to the selected “group at risk” (combined genotypes AA+AT). Observed frequencies of variance of* FTO *gene rs9939609 in patients with psoriasis, among men and women, are shown in Table 1.

### 3.2. Relationship between the Presence of A Allele, Severity of PSO, and Inflammation Markers

The mean PASI value within the carriers of AA alleles was significantly higher than in the TT group (11.96 ±6.38* vs. *10.01±8.55, p=0.02). The difference between genotype groups AA+AT and TT did not achieve statistical significance (p=0.1). Comparison of the presence of risk allele and the investigated inflammation markers has shown that the group of at least one risk allele presented higher level of CRP compared to TT homozygotes (AA + AT* vs.* TT, p=0.04). There were no statistical differences between analyzed groups of genotypes with respect to ESR rate. The results are shown in Table 2.

### 3.3. Association of A Allele Carriers with Anthropometric Measurements and Carbohydrate Parameters

Anthropometric analysis showed that the carriers of two risk alleles had significantly higher BMI (p=0.002) and hip circumference (p=0.02) with respect to the TT homozygotes. Group with at least one risk allele had significantly higher BMI (p=0.0005) and a tendency for an increased waist (p=0.05). No statistically significant differences were found in reference to age, WHR, or blood pressure between analyzed genotypes.

Statistical analysis of carbohydrate parameters showed tendency to significance for an increased insulin concentration between group AA+AT and TT (p=0.05). Differences in HOMA, FIRI, and QUICKI values did not achieve statistical significance between analyzed groups (p=0.07). There were also no differences in glucose concentration. All of the clinical characteristics and carbohydrate parameters of PSO-patients with respect to groups of genotypes are shown in Table 2.

## 4. Discussion

Psoriasis is a chronic inflammatory disease associated with increased risk of cardiovascular conditions (CVD). Many population-based studies have shown relationship between PSO and obesity, hypertension, carbohydrate intolerance, diabetes mellitus, hyperlipidemia, or metabolic syndrome. The meta-analysis of many observational studies showed higher frequency of obesity in PSO groups with respect to control group and, what is more, severe psoriasis was more often accompanied by obesity than the mild form [4]. The cause of greater amount of fatty tissue in PSO patients is probably multifactorial. Chronic inflammation, sedentary lifestyle, and calorific diet may play the main etiologic role in this condition, followed by environmental and genetic factors.

The* FTO* gene has been associated with obesity in some populations. SNPs of* FTO* gene such as rs9930609 and rs9930506 have presented the strongest association with obesity and BMI [19]. There is little knowledge about the mechanism of influence of the* FTO* gene on increased risk of obesity and metabolic disturbances in PSO patients*. Coto-Segura et al*. published data that PSO patients, homozygous for* FTO* rs9930506 G allele, had higher BMI and increased obesity risk and probable increased risk of arthritis [14].

To expand the knowledge about genetic predisposition of psoriatic patients to obesity and related conditions, we aimed to investigate this association with SNP rs9930609 of* FTO* gene. To the best of our knowledge our research is the first one showing an association of this polymorphism with obesity, some metabolic disturbances, or severity of psoriasis.

At first, we assessed the frequency of genotypes of* FTO* gene rs9930609 among group of Polish PSO patients, representing Lower Silesia region. There is some data available on the distribution of alleles of SNP rs9939609 in Polish groups. A research of 405 children, hospitalized mainly due to some cardiovascular problems and another study, and 136 women with polycystic ovary syndrome showed higher frequency of AA genotype [20, 21]. These differences may however result from different characteristic of studied groups. The data of the population-based study, performed on a larger group of the same origin of Lower Silesia, showed similar percentages of A allele carriers. In this study, 20% of patients were AA homozygotes. Moreover,* Coto-Segura et al.* showed that the prevalence of the risk allele of* FTO* rs9930506 polymorphism in patients with psoriasis was not significantly different compared to the general population from the same region [14]. It seems that PSO patients do not show an increased frequency of risk alleles of* FTO* gene. This hypothesis however requires further research on a larger population.

The next aim of our study was to assess the relationship between the presence of A allele and clinical course and severity of psoriasis. The mean PASI value among carriers of risk alleles AA was significantly higher than in the TT group, though the differences between mean PASI score in these two groups do not seem to be biologically significant (12* vs.* 10). Group with at least one risk allele was associated with significantly higher level of CRP compared to TT homozygotes. Similar results concerning relationship of CRP and* FTO* gene have been recently presented by Spanish authors [14]. It may indicate a trend of severity of inflammation among carriers of risk allele. This data, however, require confirmation on a large group with greater differences in severity of inflammation of the disease.

Numerous researchers have found the association of* FTO* gene with obesity traits and many studies confirmed higher frequency of obesity in PSO groups. There is little knowledge about influence of* FTO* gene on increased risk of obesity and metabolic disturbances in PSO patients, which became our further aim.* Coto-Segura et al.* found that PSO patients, homozygous for* FTO* rs9930506 risk allele, had higher BMI and increased obesity risk [14]. Our analysis showed that the carriers of two risk alleles of* FTO* rs9939609 have significantly higher BMI and hip circumference with respect to TT homozygotes. Patients of “group at risk” have significantly higher BMI and a tendency to an increased waist circumference.

The large population-based study of the same region of Lower Silesia showed an increased risk of obesity in A allele carriers of* FTO* rs9939609, especially among men [22]. Similar relationship of carrying risk alleles with an increased predisposition to obesity and being overweight in groups of psoriatic patients as well as in general populations leads to the conclusion that the* FTO* gene has an influence on the body weight.

We investigated also the relationship of risk allele of* FTO* rs9939609 with hypertension that is often associated with obesity. We found no significant differences in blood pressure within the groups. Similar observations were found in study population of Lower Silesia [22]. In the MONICA study patients with at least one risk allele of* FTO* gene had higher value of RR systolic pressure. However, these differences were attenuated after adjustment of BMI, which may indicate stronger relationship of* FTO* gene with BMI than with the blood pressure [10].

Next we assessed the relationship between the presence of risk allele and some parameters of carbohydrate metabolism. We showed that the “group at risk” tends to have higher insulin concentration. We noted also some slight, although not statistically significant, differences: higher values of HOMA and FIRI and lower value of QUICKI within carriers of at least one risk allele. There were no differences in glucose concentration. Our studies suggest that A allele of* FTO *gene may potentiate insulin resistance as a consequence of coexisting obesity in PSO patients.

Presented data regarding the genetic aspects of psoriasis are worth further research on large groups of patients. However, substantially, patient with psoriasis, especially with moderate to severe, form should be included in preventive care towards early detection and treatment of coexisting obesity and metabolic disorders to reduce the existing elevated risk of developing cardiovascular conditions.

## 5. Conclusion

The presence of A allele of* FTO* gene rs9939609 is associated with more severe course of psoriasis and inflammation. We found association of risk allele with increased BMI, hip and waist circumferences, and insulin concentration. Carrying the risk allele of* FTO* rs9939609 may potentiate insulin resistance as a consequence of coexisting obesity in PSO patients.

## Figures and Tables

**Table 1:** The genotype frequency of the *FTO* gene rs9939609.

Genotypes	PSO-patients n=197	PSO-women n=96	PSO-men n=101
Carriers of AA genotype	19.87%	16.7%	25.23%
Carriers of AT genotype	43.05%	44.79%	36.94%
Carriers of AA+AT genotype	62.92%	61.46%	62.17%
Carriers of TT genotype	37.08%	38.54%	37.08%

**Table 2:** Clinical characteristics and carbohydrate parameters (mean ± SD) of PSO-patients according to AA, AA+AT and TT genotypes.

Parameter	Carriers of AA genotype n=41	Carriers of AA+AT genotype n=123	Carriers of TT genotype n=74	P value^a^	P value^b^
Age (years)	49.8±15.9	51.01±14.07	49.36±14.19	0.56	0.42
BMI (kg/m^2^)	28.6±6.5	29.52±7.05	26.25±5.55	**0.002**	**0.0005**
Waist (cm)	100.87±14.76	101.71±16.07	96.34±14.28	0.14	*0.05*
Hip (cm)	104.96±8.63	105.69±10.72	101.00±9.72	**0.02**	0.06
WHR	0.95±0.09	0.96±0.09	0.95±0.09	0.82	0.53
RR_s_ (mm Hg)	137.70±20.20	135.85±20.79	133.83±24.18	0.32	0.33
RR_d_ (mm Hg)	83.65±11.07	83.87±11.17	83.01±13.39	0.62	0.63
PASI	11.96 ±6.38	10.62±7.75	10.01±8.55	**0.02**	0.10
CRP (mg/dl)	14.39±26.93	11.22±20.26	7.77±16.58	0.07	**0.04**
ESR (mm/h)	26.00±22.24	22.95±20.17	19.34±17.69	0.09	0.14
Glucose (mg/dl)	95.60±42.45	92.10±30.46	87.14±15.62	0.97	0.85
Insulin (*µ*IU/ml)	9.64±6.10	12.32±13.31	8.78±7.28	0.34	*0.05*
HOMA	2.46±3.62	2.96±3.93	1.90±1.50	0.52	0.07
FIRI	1.51±3.26	2.66±3.53	1.71±1.35	0.52	0.07
QUCIKI	0.36±0.06	0.35±0.05	0.36±0.05	0.52	0.07

^a^p value - AA *vs.* TT

^b^p value - AA + AT *vs*. TT

## Data Availability

The data used to support the findings of this study are available from the corresponding author upon request.
